# Pathogenicity of and plant immunity to soft rot pectobacteria

**DOI:** 10.3389/fpls.2013.00191

**Published:** 2013-06-11

**Authors:** Pär R. Davidsson, Tarja Kariola, Outi Niemi, E. T. Palva

**Affiliations:** Division of Genetics, Department of Biosciences, University of HelsinkiHelsinki, Finland

**Keywords:** Pectobacterium, oligogalacturonides, necrotrophs, plant hormones, cell wall-degrading enzymes, genomics, genetics, DAMPs and PAMPs

## Abstract

Soft rot pectobacteria are broad host range enterobacterial pathogens that cause disease on a variety of plant species including the major crop potato. Pectobacteria are aggressive necrotrophs that harbor a large arsenal of plant cell wall-degrading enzymes as their primary virulence determinants. These enzymes together with additional virulence factors are employed to macerate the host tissue and promote host cell death to provide nutrients for the pathogens. In contrast to (hemi)biotrophs such as *Pseudomonas*, type III secretion systems (T3SS) and T3 effectors do not appear central to pathogenesis of pectobacteria. Indeed, recent genomic analysis of several *Pectobacterium* species including the emerging pathogen *Pectobacterium wasabiae* has shown that many strains lack the entire T3SS as well as the T3 effectors. Instead, this analysis has indicated the presence of novel virulence determinants. Resistance to broad host range pectobacteria is complex and does not appear to involve single resistance genes. Instead, activation of plant innate immunity systems including both SA (salicylic acid) and JA (jasmonic acid)/ET (ethylene)-mediated defenses appears to play a central role in attenuation of *Pectobacterium* virulence. These defenses are triggered by detection of pathogen-associated molecular patterns (PAMPs) or recognition of modified-self such as damage-associated molecular patterns (DAMPs) and result in enhancement of basal immunity (PAMP/DAMP-triggered immunity or pattern-triggered immunity, PTI). In particular plant cell wall fragments released by the action of the degradative enzymes secreted by pectobacteria are major players in enhanced immunity toward these pathogens. Most notably bacterial pectin-degrading enzymes release oligogalacturonide (OG) fragments recognized as DAMPs activating innate immune responses. Recent progress in understanding OG recognition and signaling allows novel genetic screens for OG-insensitive mutants and will provide new insights into plant defense strategies against necrotrophs such as pectobacteria.

## INTRODUCTION

Plant pathogens including pathogenic bacteria use a variety of strategies ranging from stealth to brute force to colonize plants and derive nutrients from their hosts. The stealth strategy is employed by biotrophs and hemibiotrophs such as *Pseudomonas syringae* and *Xanthomonas* spp. that rely on living plant cells for nutrient acquisition at least until later stages of infection. Their lifestyle is largely dependent on their ability to avoid and suppress plant defense responses most notably by secretion of effector proteins enabling them to obtain nutrients and multiply within living plant tissue (Göhre and Robatzek, 2008; Collmer et al., 2009; Kay and Bonas, 2009). Bacterial effectors are secreted mainly through the type III secretion system (T3SS) which is a multi-protein injection machinery capable of translocating proteins directly from the bacterial cytosol into the host cell (Alfano and Collmer, 2004). Different effector proteins target specific components of plant defense and are effective only against a particular plant species or cultivar. Therefore, strains of (hemi)biotrophic bacteria often show a high degree of host specificity (Niks and Marcel, 2009; Lindeberg et al., 2012). Although essential to pathogenicity of (hemi)biotrophs, T3SS and effectors play a much less central role in the virulent lifestyle of necrotrophs. Instead, necrotrophs use a brute force strategy employing plant cell wall-degrading enzymes (PCWDEs), necrosis-inducing proteins and toxins to actively kill plant tissue and feed on the nutrients released. For example, the broad host range necrotrophic fungus *Botrytis cinerea* uses enzymes to break down the host cell walls to access the host tissue and causes host cell death by production of non-specific fungal toxins and reactive oxygen species (ROS; van Kan, 2006; Choquer et al., 2007). Similarly, bacterial necrotrophs such as soft rot pectobacteria are broad host range pathogens that are particularly effective in macerating the host tissues and obtaining nutrients from the dead cells. Recent progress in genomic analysis of several species of pectobacteria has provided new insights into the necrotrophic lifestyle of these pathogens and has also made them excellent models for elucidating the strategies and immune responses plants employ to combat bacterial necrotrophs.

The invasion of a phytopathogen triggers immune responses in the host plant. While lacking the somatic, adaptive immune system as well as mobile defender cells present in animals, plants are still capable of defending themselves in various ways. The recognition of the invader usually occurs via pathogen-associated molecular patterns (PAMPs), conserved structures such as the bacterial flagellin essential for the microbial lifestyle (Gómez-Gómez and Boller, 2000; Boller and Felix, 2009). The resulting pattern-triggered immunity (PTI) response as the first line of defense is sufficient to fend off many but not all potential pathogens. Successful pathogens can bypass PTI for example by secreting the above mentioned effector proteins that interfere with the PTI responses and hence, benefit pathogen virulence by causing effector-triggered susceptibility (ETS). More severe defense responses triggered upon effector recognition including hypersensitive response (HR) and programmed cell death (PCD) result in effector-triggered immunity (ETI) particularly effective against strains of (hemi)biotrophs (Jones and Dangl, 2006). Plants can also sense danger via recognition of so called danger or damage-associated molecular patterns (DAMPs) that report of “damage to self” and induce a variety of host defense responses in many aspects similar to those triggered by PAMPs (Ridley et al., 2001; Galletti et al., 2009). These can be fragments of plant cell wall released by the action of chewing insects but also by PCWDEs secreted by necrotrophic pathogens as an essential part of their virulence strategy. Central in mediating the innate immunity responses are phytohormones such as salicylic acid (SA), jasmonic acid (JA), and ethylene (ET; Dodds and Rathjen, 2010). The focus of this review will be on the virulence strategies of pectobacteria and the corresponding immune responses of plants addressing both similarities and differences of immune responses of pectobacteria to those of biotrophs/hemibiotrophs such as *Pseudomonas*.

## SOFT ROT PECTOBACTERIA

Soft rot enterobacteria of genera *Pectobacterium* and *Dickeya* are classical and well-studied examples of necrotrophic plant pathogenic bacteria. Their taxonomical classification has been revised several times in recent years. They were first characterized in the early 20th century (Jones, 1901) and for decades were known as members of the genus *Erwinia* (Winslow et al., 1920). In 1998, the genus was divided into three phylogenetic groups (Hauben et al., 1998). Soft rot pathogens were moved out of the genus *Erwinia* which now contains plant pathogens such as the hemibiotroph *Erwinia amylovora*. Two new genera were created: *Brenneria* and *Pectobacterium*, the latter of which harbors the soft rot species. Later on, subspecies of *P. carotovorum* were raised at species level giving rise to for example *P. wasabiae* and *P. atrosepticum* (Gardan et al., 2003). Further, *P. aroidearum* was described as a novel species consisting of soft rot pathogens mainly infecting monocotyledonous plants (Nabhan et al., 2012a). Finally, *P. chrysanthemi* was separated from *Pectobacterium* and the new genus *Dickeya* was formed (Samson et al., 2005).

Soft rot enterobacteria are the most important causative agents of the economically significant soft rot disease which results in significant crop losses during the growth season as well as during storage (Perombelon and Kelman, 1980; Pérombelon, 2002; Czajkowski et al., 2011). The most distinctive feature of soft rot pathogenesis is the co-ordinate production of a large arsenal of PCWDEs such as pectinases, cellulases, hemicellulases, and proteinases which makes pectobacteria very effective in decaying plant tissue. PCWDEs are secreted mainly through type II secretion system (T2SS; Pérombelon, 2002; Charkowski et al., 2012). In addition to PCWDEs, soft rot bacteria secrete proteins that promote plant cell death such as the necrosis-inducing protein (Nip) and the effector protein DspE (Mattinen et al., 2004; Kim et al., 2011). Typical for necrotrophs, soft rot bacteria generally display a broad host range. The disease affects several important crop and ornamental species across the world. Bacteria of the genus *Dickeya* cause disease especially in tropical and subtropical climates and the host plants include maize, banana, and increasingly potato among many other crop species (Pérombelon, 2002; Toth et al., 2003, Toth et al., 2011; Samson et al., 2005). The host range of *P. carotovorum* is considered to be the widest of all the soft rot bacteria, potato being the most important crop affected in temperate regions (Pérombelon, 2002; Toth et al., 2003). Common soft rot of potato tubers caused by *P. carotovorum* can result in extensive crop losses also post-harvest during storage (Perombelon and Kelman, 1980; Pérombelon, 2002). *P. atrosepticum*, unlike *P. carotovorum*, appears more host-specific. This pathogen causes a stem disease called blackleg on potato in temperate climates (Perombelon and Kelman, 1980; Pérombelon, 2002). The reason for the narrow host range remains to be elucidated. The third economically important *Pectobacterium* species, *P. wasabiae*, was originally characterized as a pathogen of Japanese horseradish (*Wasabia japonica*), i.e., wasabi (Goto and Matsumoto, 1987). However, recently *P. wasabiae* has received attention as a potato pathogen in several countries around the world (Ma et al., 2007; Pitman et al., 2008; Baghaee-Ravari et al., 2010). Also, strains previously characterized as *P. carotovorum* have recently been re-identified as *P. wasabiae* (Nabhan et al., 2012b) including a well-studied Finnish model strain SCC3193 (Nykyri et al., 2012). At this point, it is not known if *P. wasabiae* represents an emerging potato pathogen currently spreading around the world or if the species has long been present on potato fields but only recently sequence based methods have enabled the differentiation of *P. wasabiae* from *P. carotovorum*.

Although soft rot enterobacteria have been studied for decades, very little still is known of their survival strategies between growing seasons. Soft rot pathogens have been shown to be able to persist in soil only for weeks or months depending on environmental conditions and overwintering in soil is not considered likely. However, survival on decomposing plant material in soil is known to happen (Perombelon and Kelman, 1980; Czajkowski et al., 2011). Introduction of the soft rot pathogens via contaminated planting material such as seed tubers is considered to be the most common way for the disease to spread and considerable effort is taken to ensure disease free planting material. However, due to the ability of soft rot bacteria to colonize plants latently without symptoms, the level of control achieved varies and is highly dependent on environmental conditions (Czajkowski et al., 2011). Further, dispersal of the bacteria could also happen via usage of surface water for irrigation, via aerosols generated by rain, via movement of the bacteria in soil water or mechanically via contaminated agricultural equipment (Perombelon and Kelman, 1980; Czajkowski et al., 2011). Moreover, insects can act as vectors for many plant pathogenic bacteria (Nadarasah and Stavrinides, 2011). Soft rot enterobacteria have also been found associated with insects and transmission via insects has been suggested for decades (Perombelon and Kelman, 1980; Charkowski et al., 2012). Certain strains of *P. carotovorum* have indeed been shown to interact with *Drosophila* and activate an immune response in the fly (Basset et al., 2000). The interaction has been shown to be promoted by the bacterial gene *evf* which improves the persistence of the bacteria in the gut of the fly host (Basset et al., 2003). The existence of bacterial genes promoting interactions with insects suggests that adaptation to insects as vectors or as alternative hosts may have played an important role in the evolution of these plant pathogenic bacteria.

## GENOME ANALYSIS AND VIRULENCE FACTORS OF PECTOBACTERIA

Genome sequencing has provided new insights into the lifestyle of *Pectobacterium*. *P. atrosepticum* SCRI1043 was sequenced in 2004 as the first soft rot pathogen (Bell et al., 2004, accession: BX950851). By December 2012, seven more *Pectobacterium* genome sequences have become publicly available: *P. carotovorum* WPP14 (Glasner et al., 2008, accession: PRJNA31123), *P. brasiliensis* PBR1692 (Glasner et al., 2008, accession: PRJNA31121), *P. aroidearum* PC1 (accession: CP001657.1), *P. wasabiae* WPP163 (accession: CP001790.1), *P. wasabiae* SCC3193 (Koskinen et al., 2012, accession: CP003415), *P. wasabiae* CFBP 3304^T^ (Nykyri et al., 2012, accession: AKVS00000000), and *P. carotovorum* subsp. *carotovorum* PCC21 (Park et al., 2012, accession: CP003776).

The necrotrophic nature of the symptomatic stage of *Pectobacterium* infection and the role of PCWDEs has long been appreciated. Genomic approaches have indeed shown that different *Pectobacterium* species share a similar collection of PCWDEs instrumental for host tissue maceration (Glasner et al., 2008; Nykyri et al., 2012). The genes encoding PCWDEs are scattered around the *Pectobacterium* genomes and are mainly found from the core genome (Toth et al., 2006; Glasner et al., 2008; Nykyri et al., 2012). Apart from the similarities in the enzyme arsenal, limited strain specific differences exist for example in the composition of putative proteinases and in the lack of the pectate lyase HrpW and the polygalacturonase (PG) PehK in *P. wasabiae* strains (Nykyri et al., 2012). Consequently, differences in host specificity or disease type and severity between different *Pectobacterium* species and strains is not explained by the arsenal of PCWDEs but is likely to rely on other factors yet to be characterized.

Although the necrotrophic lifestyle of *Pectobacterium* is a hallmark of the symptomatic phase of infection, in recent years the view of *Pectobacterium* has shifted from a simple necrotroph toward a more sophisticated pathogen whose action at the early stages of infection can be better described as biotrophic (Toth and Birch, 2005; Liu et al., 2008). The initiation of soft rot disease is highly dependent on environmental conditions and the soft rot bacteria are indeed considered as opportunistic pathogens (Pérombelon, 2002; Toth and Birch, 2005). They are capable of living within the plant tissue without causing symptoms but this asymptomatic stage ends when high moisture and low oxygen concentration lower plant resistance favoring bacterial growth (Perombelon and Kelman, 1980; Pérombelon, 2002). PCWDEs are produced, and therefore the symptoms appear, only when the cell density of the bacteria is high (Perombelon and Kelman, 1980; Pérombelon, 2002; Toth et al., 2003). The production of PCWDEs is strictly controlled in a population density-dependent manner through quorum sensing (QS) regulation (Jones et al., 1993; Pirhonen et al., 1993; Liu et al., 2008). This is suggested to prevent premature activation of plant defenses as the action of PCWDEs releases cell wall fragments which trigger defense responses in the host plant (Palva et al., 1993; Salmond et al., 1995; Mäe et al., 2001). QS is proposed to function as the master switch controlling various virulence determinants to achieve a successful transition from the asymptomatic biotrophic phase to necrotrophy (Liu et al., 2008). In addition to QS, the production of PCWDEs and other virulence determinants is further controlled by a number of two component systems and other regulators which sense physiological or environmental cues such as plant derived organic acids and pectin derivatives indicative of presence of the host plant. The effect of different cellular and environmental signals is then integrated by global regulators to assure an appropriate response (reviewed in Toth et al., 2006; Charkowski et al., 2012).

The role of horizontal gene transfer in acquisition of determinants related to interaction with host plants has been highlighted in *Pectobacterium* genome studies (Bell et al., 2004; Toth et al., 2006; Glasner et al., 2008; Nykyri et al., 2012). Many of these traits could benefit the bacterium at the early stages of infection. Bell et al. (2004) identified putative virulence determinants within horizontally acquired islands in the genome of *P. atrosepticum* and experimentally verified the contribution of a putative *virB*-type type IV secretion system (T4SS) and a polyketide phytotoxin (encoded by the *cfa* cluster, see below) to virulence of the bacterium. T4SS machineries translocate DNA and/or proteins across the bacterial cell wall into bacterial or eukaryotic cells (Christie et al., 2005). *virB*-T4SS was first described in *Agrobacterium tumefaciens* where it is used to deliver the tumorigenic Ti-plasmid into the plant cell (Gelvin, 2009). The nature of the material translocated through the T4SS in *P. atrosepticum* remains unknown and the role of T4SS in other *Pectobacterium* species, where sporadically present, has not been characterized. *P. wasabiae* SCC3193 and CFBP 3304^T^ as well as *P. carotovorum* subsp. *brasiliensis* PBR1692 harbor T4SS, whereas it is absent from *P. wasabiae* WPP163, *P. carotovorum* WPP14, and *P. aroidearum* PC1 (Glasner et al., 2008; Nykyri et al., 2012). The *cfa* cluster in *P. atrosepticum* encodes enzymes for the synthesis of coronafacic acid part of the coronatine phytotoxin characterized in *P. syringae* as an important virulence determinant which acts by mimicking JA (Bender et al., 1999; Bell et al., 2004). However, coronatine is not produced by *P. atrosepticum* as enzymes for synthesis of coronamic acid, which is also required for biosynthesis of coronatine, are missing (Bell et al., 2004). Bell et al. (2004) speculate that the effect of the *cfa* cluster on virulence of *P. atrosepticum* could be through the production of an alternative polyketide phytotoxin.

The collection of horizontally acquired islands differs between *Pectobacterium* species and strains (Glasner et al., 2008; Nykyri et al., 2012). Some islands are present in all genomes studied, whereas many islands can only be identified in one strain or species. It remains to be seen if the varying collection of horizontally acquired islands is responsible for the differences in virulence and host specificity between *Pectobacterium* species. For example, the *cfa* cluster present on an island in *P. atrosepticum* is missing from the genomes of other *Pectobacterium* (Glasner et al., 2008; Nykyri et al., 2012). Further, Nykyri et al. (2012) reported the finding of several *P. wasabiae* specific islands present in all three *P. wasabiae* strains (WPP163, SCC3193, and CFBP 3304^T^) but absent from genomes of other *Pectobacterium* species. These islands contain uncharacterized genes but also genes encoding for example a second type VI secretion (T6SS) machinery and a bacterial microcompartment of unknown function. T6SS is a common protein secretion system in Gram-negative bacteria and it has been reported to function in interactions with animals, plants and other bacteria (Schwarz et al., 2010; Records, 2011; Russell et al., 2011; Zheng et al., 2011). T6SS was first shown to contribute to virulence on potato in *P. atrosepticum* (Liu et al., 2008). *P. atrosepticum* and *P. carotovorum* genomes contain only one T6SS machinery whereas *P. wasabiae* harbors two machineries of which one is similar to the T6SS in other *Pectobacterium* species and the other rather resembles the machinery in *Pantoae* and *Erwinia* (Nykyri et al., 2012). In *P. wasabiae* SCC3193, the two T6SS machineries were experimentally shown to have at least partially overlapping functions during potato infection (Nykyri et al., 2012). Effectors secreted through the T6SS have not yet been identified in *Pectobacterium* and the exact role of T6SS during infection remains to be elucidated.

## MODULATION OF HOST RESPONSES BY PECTOBACTERIA

Very little is known of the mechanisms used by *Pectobacterium* to avoid being destroyed by host defense responses during the asymptomatic phase. Most hemibiotrophic bacterial plant pathogens rely on T3SS and T3 effector proteins to manipulate their hosts in order to achieve suppression of plant defenses and mutants in T3SS are consequently non-pathogenic (Grant et al., 2006; Collmer et al., 2009). T3SS has been shown to contribute to virulence of *P. atrosepticum* and *P. carotovorum* (Rantakari et al., 2001; Holeva et al., 2004; Kim et al., 2011). Indeed, inactivation of T3SS in *P. carotovorum* resulted in delayed growth of the bacteria at the early stages of infection (Rantakari et al., 2001), suggesting that T3SS could be used to suppress plant defense responses also in *Pectobacterium*. However, the number of T3 effectors encoded by *Pectobacterium* genomes is apparently much smaller than that of hemibiotroph genomes (Glasner et al., 2008; Kim et al., 2009) and no T3 effectors suppressing plant defense responses have been described. In contrast, the only characterized T3 effector in *Pectobacterium*, DspE, elicits plant cell death which in turn promotes disease progression and maceration of plant tissue by *Pectobacterium* at the nectrotrophic stage of infection (Kim et al., 2011). It was concluded that *P. carotovorum* may not at all use T3SS to suppress plant defense responses as the gene expression profile of *Nicotiana benthamiana* after *P. carotovorum* infection is similar to that of *P. syringae* T3SS mutant rather than wild type *P. syringae*. Moreover, *P. carotovorum* was unable to suppress a typical basal defense response, callose deposition in leaves (Kim et al., 2011). This is contrary to *P. syringae* DC3000 where the corresponding T3 effector AvrE inactivates SA-dependent basal defenses (DebRoy et al., 2004). The limited role of T3SS in *Pectobacterium* virulence is further supported by studies showing that when the virulence of T3SS harboring and naturally T3SS-deficient *P. carotovorum* strains was compared, no obvious differences were observed (Kim et al., 2009). Furthermore, *P. wasabiae* seems to entirely lack T3SS (Ma et al., 2007; Kim et al., 2009; Pitman et al., 2009; Nykyri et al., 2012). Although the machinery and associated effectors may contribute to virulence, *Pectobacterium* clearly does not rely on T3SS to establish a successful infection. Other subtle mechanisms to manipulate the host at the early stages of infection must exist. For example, T6SS is hypothesized to manipulate host defense responses (Liu et al., 2008), but no T6 effectors with this ability have so far been described. It remains an open question, whether all *Pectobacterium* species use the same strategies or if each species or strain possesses its own collection of mechanisms enabling a successful interaction with the host.

One putative strategy for an effector protein or a virulence determinant to function early on in infection is to manipulate the hormonal balance of the host plant. Plant hormones are central mediators of plant growth, development, and responses to abiotic stress as well as plant defenses. Furthermore, hormonal crosstalk plays a key role in determining plant response priorities to environmental cues influencing the outcome of plant–pathogen interactions (Dong, 1998; Robert-Seilaniantz et al., 2011). Consequently, many plant pathogenic, as well as plant growth-promoting, bacteria have the ability to manipulate hormonal signaling in plants by producing plant hormones and hormone mimics or by influencing the crosstalk between hormonal pathways (Costacurta and Vanderleyden, 1995; Robert-Seilaniantz et al., 2011). Bacterial synthesis of auxin (indole-3-acetic acid), cytokinins, ET, and abscisic acid (ABA) has been reported (Robert-Seilaniantz et al., 2011). Among soft rot bacteria, only *Dickeya dadantii* 3937 has been shown to produce auxin (Yang et al., 2007). However, auxin was described to have a regulatory role in bacterial virulence gene expression and it remains to be shown whether the production also affects plant hormone signaling. The coronatine toxin produced by *P. syringae* has been shown to act as a JA mimic activating JA-dependent defenses and suppressing antagonistic SA-dependent defenses (Laurie-Berry et al., 2006; Uppalapati et al., 2007; Zheng et al., 2012). In the case of *Pectobacterium*, no direct evidence for virulence determinants affecting plant hormones exists. The *P. atrosepticum* polyketide phytotoxin encoded by the *cfa* cluster can be thought of as a potential candidate. Furthermore, Nykyri et al. (2012) reported the interesting finding of a gene encoding a putative *S*-adenosyl-L-methionine:benzoic acid/SA carboxyl methyltransferase on a horizontally acquired island in the genome of *P. wasabiae* SCC3193. This gene is also present in the genome of *P. wasabiae* WPP163 but it is absent from other *Pectobacterium* strains. In fact, the putative protein resembles plant enzymes and was concluded to be of probable eukaryotic origin. The corresponding methyltransferases in plants are involved in production of the mobile signal methyl salicylate by methylation of SA in response to pathogen attack (Ross et al., 1999; Chen et al., 2003; Park et al., 2007). The *P. wasabiae* benzoic acid/SA methyltransferase could represent a novel direct way to manipulate SA-mediated defenses instead of indirect effect through antagonistic hormonal pathways. However, this hypothesis still needs to be experimentally verified.

## PLANT INNATE IMMUNITY

Inducible plant innate immunity responses are comprised of two separate lines of defense that are distinguished by the type of pathogen-derived molecules (elicitors) recognized. The first has an equivalent in animals and is triggered after the perception of a group of conserved, or general, pathogen-derived molecules, called PAMPs/MAMPs that can be present in both pathogenic and non-pathogenic microorganisms (Parker, 2003; Jones and Dangl, 2006). Well-characterized PAMPs include bacterial flagellin, lipopolysaccharides (LPSs) and elongation factor Tu (EF-Tu) as well as chitin, a component of fungal cell walls (Boller, 1995; Gómez-Gómez and Boller, 2000; Robatzek et al., 2006) and are central for pathogen fitness (Parker, 2003; Chisholm et al., 2006; Dodds and Rathjen, 2010; Segonzac and Zipfel, 2011). Plants recognize PAMPs via specific pattern recognition receptors, PRRs. Of several known PRRs, best characterized are the *Arabidopsis* receptor kinase FLAGELLIN SENSING 2 FLS2) and EF-Tu receptor (EFR), that recognizes one of the most abundant and conserved proteins of bacteria, Ef-Tu (Gómez-Gómez and Boller, 2000; Zipfel et al., 2004; Boller and Felix, 2009). Recognition of PAMPs ultimately leads to PTI and hence, improved resistance. Independent of their lifestyle, different types of pathogens trigger plant defenses via PAMP recognition. For example both hemibiotrophic *Pseudomonas* and necrotrophic *Pectobacterium* trigger PTI responses through the recognition of flagellin (Desender et al., 2007; Dodds and Rathjen, 2010). Furthermore, similar to animals, plant immunity also relies on the ability to sense invading microbes by means of endogenous molecular patterns that are present only when plant tissue is infected or damaged (i.e., damage to self). The defense response elicited by recognition of these DAMPs, shares similar elements to that triggered by PAMPs (Boller and Felix, 2009; Zipfel and Robatzek, 2010).

The second line of inducible plant defense is activated in response to pathogen-secreted effectors that aim to suppress the PTI response triggered by PAMP/DAMP recognition (Jones and Dangl, 2006; Dodds and Rathjen, 2010). In contrast to PAMPs, effectors are characteristically variable and dispensable. The difference between effectors and PAMPs is the specificity of effector action to certain pathogen strains, mainly those with biotrophic and hemibiotrophic lifestyles (Tao et al., 2003; Dodds and Rathjen, 2010). For example individual strains of the hemibiotroph *Pseudomonas syringae* usually express 15–30 effectors depending on the strain (Lindeberg et al., 2012). Effectors target many processes in the plant cell. Examples of effector action in dampening the PTI are *P. syringae* effectors AvrPto and AvrPtoB that directly target PAMP receptors FLS2 and EFR (Lindeberg et al., 2012). Effectors can be recognized by corresponding resistance (R) proteins of the plant: either directly or through their action on host targets of the effectors (Jones and Dangl, 2006). The recognition events trigger defense responses, ETI including a local PCD, the HR that is efficient in limiting the infection of biotrophs that require living cells for nutrition (Glazebrook, 2005).

In contrast to biotrophs, necrotrophic pathogens have more aggressive and wide-ranging virulence strategies aiming for host cell death and hence, acquisition of nutrients from dead plant tissue. Some, like *Pectobacterium* secrete an extensive array of PCWDEs and others, like the fungal necrotroph *B. cinerea* rely on the secretion of toxins as main virulence factors (Mengiste, 2012). As a result of their lifestyle also plant immune response to necrotrophs is to some extent contrasting to that triggered by biotrophic pathogens. For example, HR resulting from effector-R-protein interaction would rather benefit than stop necrotrophic pathogens, since the success of their virulence relies on the capability to kill plant cells (Glazebrook, 2005; Mengiste, 2012). Thus, in contrast to biotrophs host cell death can actually be promoted by pathogens with necrotrophic lifestyle to facilitate their infection (Lai et al., 2011). Indeed, necrotrophic fungi such as *B. cinerea* has been shown to trigger dell death by producing ROS and non-specific toxins (Govrin and Levine, 2000) while other fungal necrotrophs employ host selective toxins to subvert ETI to ETS (Mengiste, 2012). Similarly, bacterial necrotrophs like *Pectobacterium* secrete necrosis-inducing proteins like Nip and putative effectors like HrpN proteins to promote cell death (Kariola et al., 2003; Mattinen et al., 2004). Consequently, PTI can be considered as the main plant defense strategy against necrotrophs like *Pectobacterium*.

## PHYTOHORMONE SIGNALING IN PLANT IMMUNITY

Interestingly, according to current knowledge, the perception of all the defense elicitors, PAMPs, DAMPs, and effectors appears to trigger similar immediate defense signaling. The difference between PTI and ETI is rather in strength and durability of the response than in quality and of these ETI is stronger and longer lasting (Tao et al., 2003; Espinosa and Alfano, 2004; Kim et al., 2005; Jones and Dangl, 2006). The defense responses are typically mediated by and dependent on the action of different phytohormones and indeed, depending on the lifestyle of the attacking pathogen, plant synthesizes one or more phytohormones to achieve the best possible defense response. The roles of SA, JA, and ET in orchestrating the main defense pathways triggered in response to different pathogens are well-established (Kunkel and Brooks, 2002; Glazebrook, 2005; Pieterse et al., 2009). SA has traditionally been thought to activate defense signaling targeted against biotrophic and hemibiotrophic pathogens while JA and ET defenses are associated with defense responses against necrotrophs (Kunkel and Brooks, 2002; Glazebrook, 2005). Although this remains broadly true, the signaling network triggered by many pathogens appears more complex; for example the combination of JA and ET signaling is efficient against the necrotroph *B. cinerea*, yet also SA appears to have a role in local immunity against this fungus (Ferrari et al., 2007). Interestingly, resistance against *Pectobacterium* can be enhanced by the induction of either JA/ET-mediated (Vidal et al., 1997; Norman-Setterblad et al., 2000) or SA-mediated (Palva et al., 1994; Li et al., 2004) defenses. This apparent controversy could be explained partly by the overlapping defenses triggered and partly by the different efficacies of the defenses induced by the two pathways at the different stages of the infection. Thus, SA-mediated defenses appear to be more efficient during the latent phase of infection, i.e., when PTI is triggered via PAMP recognition (Li et al., 2004, 2006; Kariola et al., 2005). During the necrotrophic phase of *Pectobacterium* infection secretion of massive amounts of PCWDEs results in prominent tissue maceration and release of DAMPs activating JA/ET-dependent defenses (Palva et al., 1993; Vidal et al., 1997; Norman-Setterblad et al., 2000; Brader et al., 2001).

Defense pathways influence each other through a network of regulatory interactions, and thus, plant responses to pathogens are a result of this complex hormonal crosstalk (Kunkel and Brooks, 2002; Bostock, 2005; Robert-Seilaniantz et al., 2011). Crosstalk (both synergistic and antagonistic) between the hormonal pathways is indeed central to defense signaling and in defining the response priorities. For example, SA and JA signaling interact on many levels, and this relationship is in many cases mutually antagonistic (Kunkel and Brooks, 2002; Spoel et al., 2003; Li et al., 2004; Glazebrook, 2005; Spoel and Loake, 2011). For example, synthesis of JA as well as accumulation of proteinase inhibitors in response to wounding and oligosaccharides can be inhibited by SA (Pena-Cortés et al., 1993; Doares et al., 1995). Conversely, overexpression of transcription factor *WRKY70*, a central component in SA signaling in *Arabidopsis* was followed by increased SA and decreased JA signaling resulting in enhanced resistance to the hemibiotroph *Pseudomonas* but susceptibility to the fungal necrotroph *A. brassicicola* suggesting that WRKY70 is a node of interaction between these hormonal pathways (Li et al., 2004, 2006).

While the roles of phytohormones SA, JA, and ET in orchestrating the main defense pathways are well-established, other phytohormones can modulate and influence the outcome of pathogen triggered defense signaling and there is even crosstalk between biotic and abiotic signaling (Fujita et al., 2006). ABA mediates adaptive responses to various abiotic stresses and is also central to many developmental processes (Finkelstein et al., 2002; Verslues and Zhu, 2005). The role of ABA in plant–pathogen interaction is multifaceted (Ton et al., 2009; Cao et al., 2011). Activation of ABA biosynthetic and signaling pathways promotes disease susceptibility to several plant pathogens. Many studies have demonstrated antagonism between ABA and SA signaling. Endogenous ABA accumulation induced by drought stress or ABA treatment prior to infection with a virulent *P. syringae pv tomato* resulted in necrosis and chlorosis in *Arabidopsis*, symptoms similar to susceptible infection (Mohr and Cahill, 2003). Moreover, overexpression of a negative regulator of ABA-responses, *ERD15* resulted in enhanced SA signaling and improved tolerance to *Pectobacterium* (Kariola et al., 2006; Aalto et al., 2012).

Both synergistic and antagonistic effects have been described for the interaction of ABA with JA/ET signaling. (Anderson et al., 2004; Mauch-Mani and Mauch, 2005; Adie et al., 2007). For example, disruption of AtMYC2, a positive regulator of ABA signaling, resulted in up-regulation of JA/ET-dependent gene expression. Additionally, ABA-deficient mutants were shown to have improved resistance against necrotrophic fungal pathogen *Fusarium oxysporum* in *Arabidopsis* (Anderson et al., 2004). At the same time, disruption of BOS1 (*Botrytis* *susceptible 1*), that controls several ABA- and JA-regulated genes resulted in decreased tolerance to necrotrophic pathogens but also to water deficit and salt stress (Mengiste et al., 2003).

The control of stomatal aperture and hence plant water relations is one of the processes under strict hormonal control mainly by ABA. Interestingly, *Arabidopsis* stomata also close in response to bacteria or bacterial PAMPs such as Flg22 and LPS, altering their role from being plain passive pathogen entry portals into actual components of plant innate immunity (Melotto et al., 2006). PAMP-triggered stomatal closure requires SA and ABA, and thus, the response is impaired for example in ABA biosynthesis mutants (Melotto et al., 2006). Furthermore, Melotto and colleagues demonstrated that *P. syringae* strains producing the JA-Ile mimic coronatine were able to induce stomatal re-opening (Melotto et al., 2006; Zheng and He, 2010). Intriguingly, similarly to the hemibiotroph *P. syringae,* even the necrotroph *Pectobacterium* is capable of inducing stomatal re-opening after the initial, PAMP-triggered stomatal closure in *Arabidopsis* (Po-Wen et al., 2013). Furthermore, priming of PTI response with the non-protein amino acid BABA (β-aminobutyric acid) was shown to enhance SA-dependent defenses, inhibit stomatal re-opening and hence, increase the plant tolerance to *Pectobacterium* (Po-Wen et al., 2013).

## DAMAGE-ASSOCIATED MOLECULAR PATTERNS – OLIGOGALACTURONIDES

Besides rapid recognition of PAMPS, both plants and animals need to sense endogenous molecular patterns that are released upon tissue damage. Such damage can result from wounding caused by chewing insects or herbivores, or degradation of plant cell walls by microbial enzymes (Boller and Felix, 2009). The released cell wall fragments act as DAMPs and trigger PTI. Secretion of PCWDEs is central to the virulence of many necrotrophic fungi and bacteria. The fragments of plant cell wall, cutin monomers and peptides released by the action of these enzymes act as DAMPs (Boller and Felix, 2009; Galletti et al., 2009). The released peptides include systemin found in the *Solanaceae* family that triggers a response similar to that induced by mechanical wounding (Hind et al., 2010). AtPeps of *Arabidopsis* resembles systemin and are believed to be released and bind their apoplastic receptors upon cell damage (Huffaker et al., 2006). Moreover, homologues of AtPeps have now been found in most higher plants (Huffaker et al., 2013) and have been shown to induce defense against necrotrophic pathogens (Liu et al., 2013). Similar to these peptides, oligogalacturonides (OGs) seem to act as DAMPs throughout the plant kingdom and hence, operate in an evolutionary old danger sensing system resulting in PTI even in monocots (Baker et al., 1990; Côté and Hahn, 1994; Randoux et al., 2009, 2010). OGs are biologically active carbohydrates (oligosaccharins) that are breakdown products of homogalacturonan, a major component of pectin (Côté and Hahn, 1994; Ridley et al., 2001). OGs of varying chain length with a degree of polymerization (DP) ranging from 2 to over 20 are released by PGs of both bacterial and fungal pathogens. These enzymes typically break down the non-methylated polygalacturonan component of pectin and play an important role in the infection by necrotrophs. In *P. carotovorum* the endo-polygalacturonase PehA is one of the major players carrying out this function, whereas *Dickeya dadantii* only has exo cleaving PGs (Kotoujansky, 1987; Saarilahti et al., 1990; Hugouvieux-Cotte-Pattat et al., 2001). The OGs released by the action of PCWDEs secreted by necrotrophs like *Pectobacterium* trigger typical PTI responses (OG-PTI) overlapping at least partly with those induced by PAMPs including oxidative burst, cell wall strengthening, production of phytoalexins and proteinase inhibitors as well as hormone biosynthesis (Ridley et al., 2001). PCWDs are not only secreted by necrotrophs, but also play a critical role during the colonization of plant roots by symbiotic rhizobia and it has recently been proposed that OGs play a role in *Rhizobium*-legume communications (Moscatiello et al., 2012).

## OLIGOGALACTURONIDE PERCEPTION

Although OGs were the first oligosaccharins characterized (Bishop et al., 1981; Hahn et al., 1981), the signaling pathways still largely remain to be elucidated. Indeed, it was not until quite recently that the first receptor for OGs was identified (Brutus et al., 2010). The elucidation of OG signaling has been hampered by the complexity of OG responses (Côté and Hahn, 1994): OGs are involved in control of plant growth and development as well as in plant response to pathogens. The interconnected nature of OG-PTI and plant growth and development through phytohormone regulation adds another layer of complexity to this analysis. Thus, when focusing on the role of OGs in plant–pathogen interactions it is not possible, nor even desirable, to ignore the developmental role of OGs. As an example, the first observed development-related effect of exogenously applied OGs was an inhibition of auxin-induced stem elongation in peas (Branca et al., 1988), and since then further studies have solidified the role of OGs as having an antagonistic effect on auxin signaling and enhancing cytokinin-induced vegetative shoot formation (Falasca et al., 2008). Clarifying the mechanistics behind this antagonistic role to auxin could provide a fruitful approach in elucidating the detailed role of OGs in plant–pathogen interactions (Savatin et al., 2011; Qi et al., 2012).

It has long been suspected that wall-associated kinases (WAKs) are involved in OG sensing. WAK1 and WAK2 have been shown to bind to pectin *in vitro* (Kohorn et al., 2009) and WAK1 has been shown to bind specifically to OGs (Morris et al., 1982; Decreux and Messiaen, 2005; Cabrera et al., 2008). The *in vitro* binding of OGs to WAK1 required OGs with a DP over nine subunits and more particularly it seems to require formation of a calcium-induced conformational state known as egg box dimers (Morris et al., 1982; Decreux and Messiaen, 2005; Cabrera et al., 2008). The egg box form progressively, and there seems to be two different forms of perception systems in which WAK1 can recognize these dimers (Cabrera et al., 2008). Even shorter chains OGs can form calcium-induced egg box dimers. However, unlike the dimers formed by longer chains, these are unstable and easily disrupted by competing monovalent ions.

Additional indication for the role of WAKs in OG signaling came from gene expression studies demonstrating that *WAK1* is up-regulated by OGs (Denoux et al., 2008). However, silencing of the WAK gene family resulted in lethality, probably due to their involvement in regulation of growth and development. Furthermore, redundancy between different WAKs complicated the study of these potential receptors (Brutus et al., 2010). Following up on the leads, Brutus et al recently employed a domain swap approach to verify that WAK1 indeed does act as a receptor of OGs. They created chimeric receptors of EFR and WAK1 and showed that the WAK1 ectodomain could be triggered by long chain OGs to activate the EFR kinase domain, and vice versa the EFR ectodomain could be triggered by elf18 peptide to activate the WAK1 kinase domain, resulting in a defense response mimicking a normal OG response. Furthermore, WAK1 overexpressing plants were seen to be more resistant to *B. cinerea* (Brutus et al., 2010). These studies indeed suggest that WAKs could be PRRs that are involved in OG perception but do not rule out the presence of other receptor-like kinases (RLKs) involved in monitoring the cell wall integrity. Such potential candidates include for example the potato RLKs responsive to short OGs and *Pectobacterium* (Montesano et al., 2001).

## OLIGOGALACTURONIDE SIGNALING

Oligogalacturonides have been shown to rapidly stimulate an increase in cytosolic calcium (Messiaen et al., 1993; Hu et al., 2004) and production of ROS, in a wide array of different plant species (Ridley et al., 2001). Other OG-PTI responses include; expression of proteinase inhibitors, induction of phenylalanine ammonia-lyase (PAL) leading to production of phytoalexins, peroxidases, glucosinolates, lignin, production of chitinase and β-1,3-glucanase, as well as increased expression of PG-inhibiting proteins (PGIPs; reviewed in Côté and Hahn, 1994; Ridley et al., 2001). OGs induce a very strong AtRBOHD-dependent apoplastic ROS burst in *Arabidopsis* (Galletti et al., 2008). However, this oxidative burst appears not to be required for OG-induced resistance against *B. cinerea* nor expression of several OG marker genes; PAD3, AtPGIP1, RetOx, CYP 81F2, and AtWRKY40. Also it would seem that induced callose deposition does not play a major role in basal or elicitor-induced resistance to *B. cinerea* (Galletti et al., 2008).

Binding of OGs to WAK1 and most biological responses appear to require longer chain OGs (Brutus et al., 2010). However, there are a number of studies that indicate plant responses to short chain OGs. Such responses include induction of genes involved in JA biosynthesis (Norman et al., 1999) in *Arabidopsis*, induction of ET production (O’Donnell et al., 1996; Simpson et al., 1998) and production of proteinase inhibitors (Thain et al., 1990; Moloshok et al., 1992; O’Donnell et al., 1996) and depolarization of leaf mesophyll cells (Thain et al., 1990), induction of RLKs (Montesano et al., 2001) and induction of resistance against *P. carotovorum* (Weber et al., 1996; Wegener et al., 1996) in potato. Also short OGs have been seen to have a developmental effect by increasing the shoot and leaf number in strawberry plants (Miranda et al., 2007). In summary, although recent studies indicate a requirement of longer chain OGs, the specific role of shorter OGs as elicitors of PTI and developmental responses remains to be clarified. One could speculate that shorter OGs play a larger role in resistance against bacterial necrotrophs and herbivores whereas longer OGs play a more significant role against necrotrophic fungi.

Polygalacturonase-inhibiting proteins are among the OG-PTI-induced proteins produced in response to fungal necrotrophs and act directly as a defense protein by reducing the hydrolytic activity of fungal PGs, but also by delaying the breakdown by PGs they increase the formation of longer chain OGs thought to be more biologically active (De Lorenzo et al., 2001; Decreux and Messiaen, 2005).The role of PGIPs in defense against pathogens has mainly been studied using fungi, such as for example *B. cinerea* (De Lorenzo et al., 2001). However, recent studies have identified PGIP as a potentially important player also in plant defense against bacteria: PGIPs were seen to play a role in resistance of Chinese cabbage against *P. carotovorum ssp. carotovorum* (Hwang et al., 2010) and PGIPS from tomato where shown to inhibit PGs from *Ralstonia solanacearum* (Schacht et al., 2011).

Several studies have characterized the reprogramming of the transcriptome in response to OGs using microarrays of *Arabidopsis* exposed to exogenous long chain (DP 10–15) OGs and also compared the expression changes between OG-PTI and Flg22-PTI (Moscatiello et al., 2006; Ferrari et al., 2007; Denoux et al., 2008). The first genome wide transcriptome analysis OG responses employed mesophyll cell suspension cultures and focused on elucidating calcium-dependent and independent signaling pathways (Moscatiello et al., 2006). The study showed that OG-induced activation of genes involved in ET signaling required both pathways, whereas activation of JA-responsive genes appeared mainly calcium-dependent, in agreement with an earlier study (Hu et al., 2003). Further it would seem that protein kinase-dependent phosphorylation is involved in the early stages of OG signaling (Moscatiello et al., 2006). Taking a slightly different approach Ferrari et al. (2007) compared OG responses with responses to infection with *B. cinerea*. The results indicated that approximately 50% of all genes were similarly regulated upon both treatments. OG-induced resistance to *B. cinerea* was found to be independent of JA, ET, and SA signaling and dependent on PAD3. Further it was shown that both Flg22 and OGs induced resistance to *B. cinerea*. As seen previously for AtPGIP1 (Ferrari et al., 2003), PAD3 was induced independently of JA, ET, and SA. This approach was further expanded trying to elucidate the similarities and dissimilarities in response to exogenous OGs and Flg22 (Denoux et al., 2008). In general, the defense response triggered by the DAMP (OG) and the PAMP (Flg22) were quite similar. Both responses were seen to be fast and transient, with a high degree of overlap especially at shorter time points. Responses to Flg22 were generally stronger, both in number of genes and expression levels. Both Flg22 and OGs were found to activate multiple components of ET, JA, and SA pathways. Noticeably several SA-dependent genes in general, and *PR1* in particular, were found to be significantly induced only by Flg22 but not with long chain OGs, even after extended exposure. This is in contrast to an earlier study characterizing *Arabidopsis* response to mixed length OGs and showing calcium and H_2_O_2_-dependent induction of several defense-related marker genes; *CHS*, *GST*, *PAL*, and noticeably *PR1* (Hu et al., 2004). In conclusion the comparison of OG- and Flg22-triggered responses suggest that DAMP-PTI might rely more on the JA/ET-dependent signaling, in agreement with several studies of *Pectobacterium* (Palva et al., 1993; Doares et al., 1995; Vidal et al., 1997; Norman et al., 1999; Norman-Setterblad et al., 2000). This is logical, since jasmonates and other oxylipins have central role in defense responses following tissue damage and have been proposed to mediate the induction of defense in response to OG signals generated by pathogen or herbivore attacks (Farmer and Ryan, 1992).

Interestingly, recent studies indicate participation of NO in OG-PTI (Rasul et al., 2012). It was demonstrated that exogenous OGs trigger calcium and nitrate reductase-dependent NO production in *Arabidopsis*. Further, NO was found to adjust AtRBOHD-mediated ROS production, as well as regulation of OG responsive *genes* (PER4 and a β-1,3-glucanase). Furthermore, NO was found to contribute to OG-induced immunity against *B. cinerea*. Whether this applies to *Pectobacterium* remains to be demonstrated.

## SUMMARY

**FIGURE 1:**
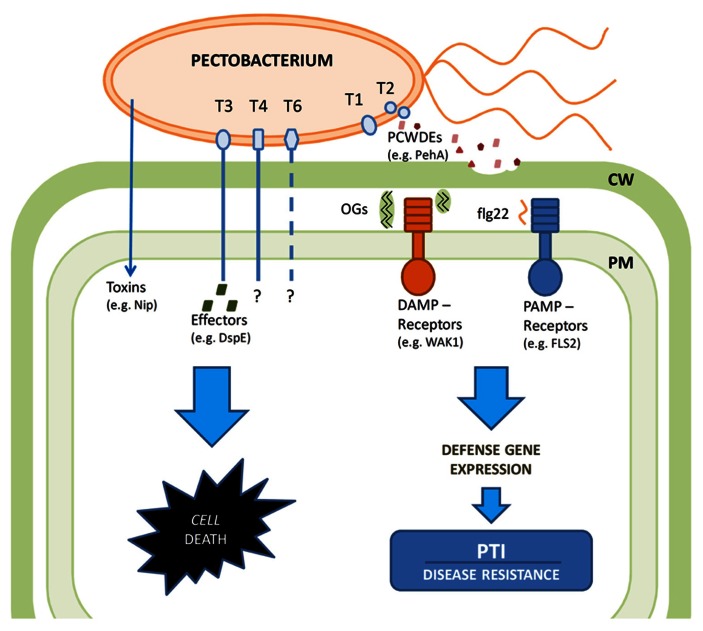
**Schematic diagram of the interactions between the bacterial necrotroph *Pectobacterium* and its host plants**. *Pectobacterium* virulence relies on macerating plant tissue through the action of PCWDEs secreted by the type I (T1) and type II (T2) secretion systems. Plant cell death is promoted by the action of toxins such as Nip and the effector DspE, which is secreted though the type III (T3) secretion system. Type IV (T4) secretion system and type VI (T6) secretion system may contribute to virulence. Plant immune responses are triggered by recognition of conserved pathogen-associated molecular patterns (PAMPs) such as flagella or damage-associated molecular patterns (DAMPs) such as OGs released by the action of PCWDEs by respective pattern recognition receptors. These recognition events in turn trigger partly overlapping defense responses including induction of defense gene expression and synthesis of various defensive compounds such as phytoalexins, defensins and PR-proteins – resulting in PTI. Bacteria can attenuate PTI particularly in the early phase of infection by tight control of PCWDE production minimizing DAMP generation. The defenses are overwhelmed at later stages by promotion of cell death and massive PCWDE production at high bacterial cell densities.

In summary, see **Figure 1**, PTI appears central to plant defense against broad host range bacterial necrotrophs like *Pectobacterium*. ETI, which is highly efficient against (hemi)biotrophs such as *Pseudomonas* is not an effective strategy to combat necrotroph infection, as ETI relies on localized cell death to trigger the downstream defense responses. Necrotrophs like *Pectobacterium* employ induction of cell death as part of their virulence strategy, thus ETI would rather enhance than prevent the infection. Indeed *Pectobacterium* species have a very limited arsenal of T3 effectors and those few that have been studied (e.g., DspE) appear to promote infection by causing cell death. Consequently, plant immune responses are triggered by recognition of conserved pathogen-associated molecular patterns (PAMPs) such as flagella or damage-associated molecular patterns (DAMPs) such as OGs released by the action of PCWDEs by respective pattern recognition receptors. While the PTI induced by PAMP recognition is a common response to both biotrophs and necrotrophs, DAMPs are more typical to necrotrophic interactions. Consequently, *Pectobacterium* strives to attenuate PTI particularly in the early phase of infection by tight control of PCWDE production minimizing DAMP generation. PAMP and DAMP recognition events trigger partly overlapping defense responses including induction of defense gene expression and synthesis of various defensive compounds such as phytoalexins, defensins, and PR-proteins – resulting in PTI. Indeed prior induction of either response will enhance plant resistance to *Pectobacterium*. Elucidating the molecular details of these two partially redundant signal networks is essential for our understanding of the plant-necrotroph interactions and can take advantage of the rapidly developing genomic techniques including transcriptional profiling and RNA sequencing combined with the powerful genetic screens available in *Arabidopsis* for mutants altered in their PTI responses. In particular elucidation of the less well-characterized OG-induced PTI deserves further studies including correlation of the chain length of the OG elicitor to a particular response at specific stages of infection and defining the downstream components that are most significant for bacterial resistance. Such studies would be crucial for providing new insights into plant defense strategies against necrotrophs. Further genome level analysis would also help to elucidate the interactions of *Pectobacterium* with other plant associated microbes, as well as their insect vectors.

## Conflict of Interest Statement

The authors declare that the research was conducted in the absence of any commercial or financial relationships that could be construed as a potential conflict of interest.

## References

[B1] Aalto M. K. Helenius E. Kariola T. Pennanen V. Heino P. Hñrak H. (2012). ERD15–an attenuator of plant ABA responses and stomatal aperture. *Plant Sci.* 182 19–28 10.1016/j.plantsci.2011.08.009 22118612

[B2] Adie B. A. T. Pérez-Pérez J. Pérez-Pérez M. M. Godoy M. Sánchez-Serrano J.-J. Schmelz E. A. (2007). ABA is an essential signal for plant resistance to pathogens affecting JA biosynthesis and the activation of defenses in *Arabidopsis*. *Plant Cell* 19 1665–1681 10.1105/tpc.106.048041 17513501 PMC1913739

[B3] Alfano J. R. Collmer A. (2004). Type III secretion system effector proteins: double agents in bacterial disease and plant defense. *Annu. Rev. Phytopathol.* 42 385–414 10.1146/annurev.phyto.42.040103.110731 15283671

[B4] Anderson J. P. Badruzsaufari E. Schenk P. M. Manners J. M. Desmond O. J. Ehlert C. (2004). Antagonistic interaction between abscisic acid and jasmonate-ethylene signaling pathways modulates defense gene expression and disease resistance in *Arabidopsis*. *Plant Cell* 16 3460–3479 10.1105/tpc.104.025833 15548743 PMC535886

[B5] Baghaee-Ravari S. Rahimian H. Shams-Bakhsh M. Lopez-Solanilla E. Antúnez-Lamas M. Rodríguez-Palenzuela P. (2010). Characterization of *Pectobacterium* species from Iran using biochemical and molecular methods. *Eur. J. Plant Pathol.* 129 413–425 10.1007/s10658-010-9704-z

[B6] Baker C. J. Mock N. Atkinson M. M. Hutcheson S. (1990). Inhibition of the hypersensitive response in tobacco by pectate lyase digests of cell wall and of polygalacturonic acid. *Physiol. Mol. Plant Pathol.* 37 155–167 10.1016/0885-5765(90)90008-L

[B7] Basset A. Khush R. S. Braun A. Gardan L. Boccard F. Hoffmann J. A. (2000). The phytopathogenic bacteria *Erwinia carotovora* infects *Drosophila* and activates an immune response. *Proc. Natl. Acad. Sci. U.S.A.* 97 3376–3381 10.1073/pnas.97.7.3376 10725405 PMC16247

[B8] Basset A. Tzou P. Lemaitre B. Boccard F. (2003). A single gene that promotes interaction of a phytopathogenic bacterium with its insect vector, *Drosophila* melanogaster. E*MBO Rep.* 4 205–209 10.1038/sj.embor.embor730 12612613 PMC1315828

[B9] Bell K. S. Sebaihia M. Pritchard L. Holden M. T. G. Hyman L. J. Holeva M. C. (2004). Genome sequence of the enterobacterial phytopathogen *Erwinia carotovora* subsp. atroseptica and characterization of virulence factors. *Proc. Natl. Acad. Sci. U.S.A.* 101 11105–11110 10.1073/pnas.0402424101 15263089 PMC503747

[B10] Bender C. L. Alarcón-Chaidez F. Gross D. C. (1999). *Pseudomonas syringae* phytotoxins: mode of action, regulation, and biosynthesis by peptide and polyketide synthetases. *Microbiol. Mol. Biol. Rev.* 63 266–292 10357851 10.1128/mmbr.63.2.266-292.1999 PMC98966

[B11] Bishop P. D. Makus D. J. Pearce G. Ryan C. A. (1981). Proteinase inhibitor-inducing factor activity in tomato leaves resides in oligosaccharides enzymically released from cell walls. *Proc. Natl. Acad. Sci. U.S.A.* 78 3536–3540 10.1073/pnas.78.6.3536 16593033 PMC319604

[B12] Boller T. (1995). Chemoperception of microbial signals in plant cells. *Annu. Rev. Plant Physiol. Plant Mol. Biol.* 46 189–214 10.1146/annurev.pp.46.060195.001201

[B13] Boller T. Felix G. (2009). A renaissance of elicitors: perception of microbe-associated molecular patterns and danger signals by pattern-recognition receptors. *Annu. Rev. Plant Biol.* 60 379–406 10.1146/annurev.arplant.57.032905.105346 19400727

[B14] Bostock R. M. (2005). Signal crosstalk and induced resistance: straddling the line between cost and benefit. *Annu. Rev. Phytopathol.* 43 545–580 10.1146/annurev.phyto.41.052002.095505 16078895

[B15] Brader G. Tas É Palva E. T. (2001). Jasmonate-dependent induction of indole glucosinolates in *Arabidopsis* by culture filtrates of the nonspecific pathogen *Erwinia carotovora*. *Plant Physiol.* 126 849–860 10.1104/pp.126.2.849 11402212 PMC111174

[B16] Branca C. De Lorenzo G. Cervone F. (1988). Competitive inhibition of the auxin-induced elongation by α-D-oligogalacturonides in pea stem segments. *Physiol. Plant.* 72 499–504 10.1111/j.1399-3054.1988.tb09157.x

[B17] Brutus A. Sicilia F. Macone A. Cervone F. De Lorenzo G. (2010). A domain swap approach reveals a role of the plant wall-associated kinase 1 (WAK1) as a receptor of oligogalacturonides. *Proc. Natl. Acad. Sci. U.S.A.* 107 9452–9457 10.1073/pnas.1000675107 20439716 PMC2889104

[B18] Cabrera J. C. Boland A. Messiaen J. Cambier P. Van Cutsem P. (2008). Egg box conformation of oligogalacturonides: the time-dependent stabilization of the elicitor-active conformation increases its biological activity. *Glycobiology* 18 473–482 10.1093/glycob/cwn027 18381977

[B19] Cao F. Y. Yoshioka K. Desveaux D. (2011). The roles of ABA in plant–pathogen interactions. *J. Plant Res.* 124 489–499 10.1007/s10265-011-0409-y 21380629

[B20] Charkowski A. Blanco C. Condemine G. Expert D. Franza T. Hayes C. (2012). The role of secretion systems and small molecules in soft-rot enterobacteriaceae pathogenicity. *Annu. Rev. Phytopathol.* 50 425–449 10.1146/annurev-phyto-081211-173013 22702350

[B21] Chen F. D’Auria J. C. Tholl D. Ross J. R. Gershenzon J. Noel J. P. (2003). An *Arabidopsis thaliana* gene for methylsalicylate biosynthesis, identified by a biochemical genomics approach, has a role in defense. *Plant J.* 36 577–588 10.1046/j.1365-313X.2003.01902.x 14617060

[B22] Chisholm S. T. Coaker G. Day B. Staskawicz B. J. (2006). Host–microbe interactions: shaping the evolution of the plant immune response. *Cell* 124 803–814 10.1016/j.cell.2006.02.008 16497589

[B23] Choquer M. Fournier E. Kunz C. Levis C. Pradier J.-M. Simon A. (2007). *Botrytis cinerea* virulence factors: new insights into a necrotrophic and polyphageous pathogen. *FEMS Microbiol. Lett.* 277 1–10 10.1111/j.1574-6968.2007.00930.x 17986079

[B24] Christie P. J. Atmakuri K. Krishnamoorthy V. Jakubowski S. Cascales E. (2005). Biogenesis, architecture, and function of bacterial type IV secretion systems. *Annu. Rev. Microbiol.* 59 451–485 10.1146/annurev.micro.58.030603.123630 16153176 PMC3872966

[B25] Collmer A. Schneider D. J. Lindeberg M. (2009). Lifestyles of the effector rich: genome-enabled characterization of bacterial plant pathogens. *Plant Physiol.* 150 1623–1630 10.1104/pp.109.140327 19515788 PMC2719148

[B26] Costacurta A. Vanderleyden J. (1995). Synthesis of phytohormones by plant-associated bacteria. *Crit. Rev. Microbiol.* 21 1–18 10.3109/10408419509113531 7576148

[B27] Côté F. Hahn M. G. (1994). Oligosaccharins: structures and signal transduction. *Plant Mol. Biol.* 26 1379–1411 10.1007/BF00016481 7858196

[B28] Czajkowski R. Pérombelon M. C. M. van Veen J. A. van der Wolf J. M. (2011). Control of blackleg and tuber soft rot of potato caused by *Pectobacterium* and *Dickeya* species: a review. *Plant Pathology* 60 999–1013 10.1111/j.1365-3059.2011.02470.x

[B29] DebRoy S. Thilmony R. Kwack Y.-B. Nomura K. He S. Y. (2004). A family of conserved bacterial effectors inhibits salicylic acid-mediated basal immunity and promotes disease necrosis in plants. *Proc. Natl. Acad. Sci. U.S.A.* 101 9927–9932 10.1073/pnas.0401601101 15210989 PMC470775

[B30] Decreux A. Messiaen J. (2005). Wall-associated kinase WAK1 interacts with cell wall pectins in a calcium-induced conformation. *Plant Cell Physiol.* 46 268–278 10.1093/pcp/pci026 15769808

[B31] De Lorenzo G. D’Ovidio R. Cervone F. (2001). The role of polygalacturonase-inhibiting proteins (PGIPs) in defense against pathogenic fungi. *Annu. Rev. Phytopathol.* 39 313–335 10.1146/annurev.phyto.39.1.313 11701868

[B32] Denoux C. Galletti R. Mammarella N. Gopalan S. Werck D. De Lorenzo G. (2008). Activation of defense response pathways by OGs and Flg22 elicitors in *Arabidopsis* seedlings. *Mol. Plant* 1 423–445 10.1093/mp/ssn019 19825551 PMC2954645

[B33] Desender S. Andrivon D. Val F. (2007). Activation of defence reactions in *Solanaceae*: where is the specificity? *Cell. Microbiol.* 9 21–30 10.1111/j.1462-5822.2006.00831.x 17081191

[B34] Doares S. H. Syrovets T. Weiler E. W. Ryan C. A. (1995). Oligogalacturonides and chitosan activate plant defensive genes through the octadecanoid pathway. *Proc. Natl. Acad. Sci. U.S.A.* 92 4095–4098 10.1073/pnas.92.10.4095 11607534 PMC41892

[B35] Dodds P. N. Rathjen J. P. (2010). Plant immunity: towards an integrated view of plant–pathogen interactions. *Nat. Rev. Genet.* 11 539–548 10.1038/nrg2812 20585331

[B36] Dong X. (1998). SA, JA, ethylene, and disease resistance in plants. *Curr. Opin. Plant Biol.* 1 316–323 10.1016/1369-5266(88)80053-0 10066607

[B37] Espinosa A. Alfano J. R. (2004). Disabling surveillance: bacterial type III secretion system effectors that suppress innate immunity. *Cell. Microbiol.* 6 1027–1040 10.1111/j.1462-5822.2004.00452.x 15469432

[B38] Falasca G. Capitani F. Della Rovere F. Zaghi D. Franchin C. Biondi S. (2008). Oligogalacturonides enhance cytokinin-induced vegetative shoot formation in tobacco explants, inhibit polyamine biosynthetic gene expression, and promote long-term remobilisation of cell calcium. *Planta* 227 835–852 10.1007/s00425-007-0660-6 17992537

[B39] Farmer E. E. Ryan C. A. (1992). Octadecanoid precursors of jasmonic acid activate the synthesis of wound-inducible proteinase inhibitors. *Plant Cell* 4 129–134 10.1105/tpc.4.2.129 12297644 PMC160114

[B40] Ferrari S. Galletti R. Denoux C. De Lorenzo G. Ausubel F. M. Dewdney J. (2007). Resistance to *Botrytis cinerea* induced in *Arabidopsis* by elicitors is independent of salicylic acid, ethylene, or jasmonate signaling but requires PHYTOALEXIN DEFICIENT3. *Plant Physiol.* 144 367–379 10.1104/pp.107.095596 17384165 PMC1913806

[B41] Ferrari S. Vairo D. Ausubel F. M. Cervone F. De Lorenzo G. (2003). Tandemly duplicated Arabidopsis genes that encode polygalacturonase-inhibiting proteins are regulated coordinately by different signal transduction pathways in response to fungal infection. *Plant Cell.* 15 93–106 10.1105/tpc.005165 12509524 PMC143454

[B42] Finkelstein R. R. Gampala S. S. L. Rock C. D. (2002). Abscisic acid signaling in seeds and seedlings. *Plant Cell* 14 S15–S45 10.1105/tpc.010441 12045268 PMC151246

[B43] Fujita M. Fujita Y. Noutoshi Y. Takahashi F. Narusaka Y. Yamaguchi-Shinozaki K. (2006). Crosstalk between abiotic and biotic stress responses: a current view from the points of convergence in the stress signaling networks. *Curr. Opin. Plant Biol.* 9 436–442 10.1016/j.pbi.2006.05.014 16759898

[B44] Galletti R. De Lorenzo G. Ferrari S. (2009). Host-derived signals activate plant innate immunity. *Plant Signal. Behav.* 4 33–34 10.4161/psb.4.1.7224 19704701 PMC2634066

[B45] Galletti R. Denoux C. Gambetta S. Dewdney J. Ausubel F. M. De Lorenzo G. (2008). The AtrbohD-mediated oxidative burst elicited by oligogalacturonides in *Arabidopsis* is dispensable for the activation of defense responses effective against *Botrytis cinerea*. *Plant Physiol.* 148 1695–1706 10.1104/pp.108.127845 18790995 PMC2577270

[B46] Gardan L. Gouy C. Christen R. Samson R. (2003). Elevation of three subspecies of *Pectobacterium carotovorum* to species level: *Pectobacterium atrosepticum* sp. nov. *Pectobacterium betavasculorum* sp. nov. and *Pectobacterium wasabiae* sp. nov. *Int. J. Syst. Evol. Microbiol.* 53 381–391 10.1099/ijs.0.02423-0 12710602

[B47] Gelvin S. B. (2009). *Agrobacterium* in the genomics age. *Plant Physiol.* 150 1665–1676 10.1104/pp.109.139873 19439569 PMC2719113

[B48] Glasner J. D. Marquez-Villavicencio M. Kim H.-S. Jahn C. E. Ma B. Biehl B. S. (2008). Niche-specificity and the variable fraction of the *Pectobacterium* pan-genome. *Mol. Plant Microbe Interact.* 21 1549–1560 10.1094/MPMI-21-12-1549 18986251

[B49] Glazebrook J. (2005). Contrasting mechanisms of defense against biotrophic and necrotrophic pathogens. *Annu. Rev. Phytopathol.* 43 205–227 10.1146/annurev.phyto.43.040204.135923 16078883

[B50] Gómez-Gómez L. Boller T. (2000). FLS2: an LRR receptor-like kinase involved in the perception of the bacterial elicitor flagellin in *Arabidopsis*. *Mol. Cell* 5 1003–1011 10.1016/S1097-2765(00)80265-8 10911994

[B51] Goto M. Matsumoto K. (1987). *Erwinia carotovora* subsp. *wasabiae* subsp. nov. isolated from diseased rhizomes and fibrous roots of Japanese horseradish**(*Eutrema wasabi* Maxim.). *Int. J. Syst. Bacteriol.* 37 130–135 10.1099/00207713-37-2-130

[B52] Govrin E. M. Levine A. (2000). The hypersensitive response facilitates plant infection by the necrotrophic pathogen *Botrytis cinerea*. *Curr. Biol.* 10 751–757 10.1016/S0960-9822(00)00560-1 10898976

[B53] Göhre V. Robatzek S. (2008). Breaking the barriers: microbial effector molecules subvert plant immunity. *Annu. Rev. Phytopathol.* 46 189–215 10.1146/annurev.phyto.46.120407.110050 18422429

[B54] Grant S. R. Fisher E. J. Chang J. H. Mole B. M. Dangl J. L. (2006). Subterfuge and manipulation: type III effector proteins of phytopathogenic bacteria. *Annu. Rev. Microbiol.* 60 425–449 10.1146/annurev.micro.60.080805.142251 16753033

[B55] Hahn M. G. Darvill A. G. Albersheim P. (1981). Host–pathogen interactions 1. *Plant Physiol.* 68 1161–1169 10.1104/pp.68.5.1161 16662068 PMC426062

[B56] Hauben L. Moore E. R. Vauterin L. Steenackers M. Mergaert J. Verdonck L. (1998). Phylogenetic position of phytopathogens within the Enterobacteriaceae. *Syst. Appl. Microbiol.* 21 384–397 10.1016/S0723-2020(98)80048-9 9779605

[B57] Hind S. R. Malinowski R. Yalamanchili R. Stratmann J. W. (2010). Tissue-type specific systemin perception and the elusive systemin receptor. *Plant Signal. Behav.* 5 42–44 10.4161/psb.5.1.10119 20592806 PMC2835955

[B58] Holeva M. C. Bell K. S. Hyman L. J. Avrova A. O. Whisson S. C. Birch P. R. J. (2004). Use of a pooled transposon mutation grid to demonstrate roles in disease development for *Erwinia carotovora* subsp. atroseptica putative type III secreted effector (DspE/A) and helper (HrpN) proteins. *Mol. Plant Microbe Interact.* 17 943–950 10.1094/MPMI.2004.17.9.943 15384484

[B59] Hu X. Neill S. Cai W. Tang Z. (2003). Hydrogen peroxide and jasmonic acid mediate oligogalacturonic acid-induced saponin accumulation in suspension-cultured cells of *Panax ginseng*. *Physiol. Plant.* 118 414–421 10.1034/j.1399-3054.2003.00124.x

[B60] Hu X. Y. Neill S. J. Cai W. M. Tang Z. C. (2004). Induction of defence gene expression by oligogalacturonic acid requires increases in both cytosolic calcium and hydrogen peroxide in *Arabidopsis thaliana*. *Cell Res.* 14 234–240 10.1038/sj.cr.7290224 15225417

[B61] Huffaker A. Pearce G. Ryan C. A. (2006). An endogenous peptide signal in *Arabidopsis* activates components of the innate immune response. *Proc. Natl. Acad. Sci. U.S.A.* 103 10098–10103 10.1073/pnas.0603727103 16785434 PMC1502512

[B62] Huffaker A. Pearce G. Veyrat N. Erb M. Turlings T. C. J. Sartor R. (2013). Plant elicitor peptides are conserved signals regulating direct and indirect antiherbivore defense. *Proc. Natl. Acad. Sci. U.S.A.* 110 5707–5712 10.1073/pnas.1214668110 23509266 PMC3619339

[B63] Hugouvieux-Cotte-Pattat N. Blot N. Reverchon S. (2001). Identification of TogMNAB, an ABC transporter which mediates the uptake of pectic oligomers in *Erwinia chrysanthemi* 3937. *Mol. Microbiol.* 41 1113–1123 10.1046/j.1365-2958.2001.02564.x 11555291

[B64] Hwang B. H. Bae H. Lim H.-S. Kim K. B. Kim S. J. Im M.-H. (2010). Overexpression of polygalacturonase-inhibiting protein 2 (PGIP2) of Chinese cabbage (*Brassica rapa* ssp. *pekinensis*) increased resistance to the bacterial pathogen *Pectobacterium carotovorum* ssp. *carotovorum.* *Plant Cell Tissue Organ Cult* 103 293–305 10.1007/s11240-010-9779-4

[B65] Jones J. D. G. Dangl J. L. (2006). The plant immune system. *Nature* 444 323–329 10.1038/nature05286 17108957

[B66] Jones L. R. (1901). *Bacillus carotovorusn*. sp., die Ursacheeinerweichen Fäulnis der Möhre *Centralblatt Bakteriol*. *Parasitenkd. Infekt Hyg.* 2 12–21

[B67] Jones S. Yu B. Bainton N. J. Birdsall M. Bycroft B. W. Chhabra S. R. (1993). The lux autoinducer regulates the production of exoenzyme virulence determinants in *Erwinia carotovora* and *Pseudomonas aeruginosa*. *EMBO J.* 12 2477–2482 8508773 10.1002/j.1460-2075.1993.tb05902.x PMC413484

[B68] Kariola T. Brader G. Helenius E. Li J. Heino P. Palva E. T. (2006). EARLY RESPONSIVE TO DEHYDRATION 15, a negative regulator of abscisic acid responses in *Arabidopsis*. *Plant Physiol.* 142 1559–1573 10.1104/pp.106.086223 17056758 PMC1676049

[B69] Kariola T. Brader G. Li J. Palva E. T. (2005). Chlorophyllase 1, a damage control enzyme, affects the balance between defense pathways in plants. *Plant Cell* 17 282–294 10.1105/tpc.104.025817 15598807 PMC544505

[B70] Kariola T. Palomäki T. A. Brader G. Palva E. T. (2003). *Erwinia carotovora* subsp. *carotovora* and *Erwinia*-derived elicitors HrpN and PehA trigger distinct but interacting defense responses and cell death in *Arabidopsis. Mol. Plant Microbe Interact.* 16 179–187 10.1094/MPMI.2003.16.3.179 12650449

[B71] Kay S. Bonas U. (2009). How *Xanthomonas* type III effectors manipulate the host plant. *Curr. Opin. Microbiol.* 12 37–43 10.1016/j.mib.2008.12.006 19168386

[B72] Kim H.-S. Ma B. Perna N. T. Charkowski A. O. (2009). Phylogeny and virulence of naturally occurring type III secretion system-deficient Pectobacterium strains. *Appl. Environ. Microbiol.* 75 4539–4549 10.1128/AEM.01336-08 19411432 PMC2704834

[B73] Kim H.-S. Thammarat P. Lommel S. A. Hogan C. S. Charkowski A. O. (2011). Pectobacterium carotovorum elicits plant cell death with DspE/F but the *P. carotovorum* DspE does not suppress callose or induce expression of plant genes early in plant–microbe interactions. *Mol. Plant Microbe Interact.* 24 773–786 10.1094/MPMI-06-10-0143 21469936

[B74] Kim M. G. da Cunha L. McFall A. J. Belkhadir Y. DebRoy S. Dangl J. L. (2005). Two *Pseudomonas syringae* type III effectors inhibit RIN4-regulated basal defense in *Arabidopsis*. *Cell* 121 749–759 10.1016/j.cell.2005.03.025 15935761

[B75] Kohorn B. D. Johansen S. Shishido A. Todorova T. Martinez R. Defeo E. (2009). Pectin activation of MAP kinase and gene expression is WAK2 dependent. *Plant J.* 60 974–982 10.1111/j.1365-313X.2009.04016.x 19737363 PMC3575133

[B76] Koskinen J. P. Laine P. Niemi O. Nykyri J. Harjunpää H. Auvinen P. (2012). Genome sequence of *Pectobacterium* sp. Strain SCC3193. *J. Bacteriol.* 194 6004 10.1128/JB.00681-12 PMC3486080 23045508

[B77] Kotoujansky A. (1987). Molecular genetics of pathogenesis by soft-rot Erwinias. *Annu. Rev. Phytopathol.* 25 405–430 10.1146/annurev.py.25.090187.002201

[B78] Kunkel B. N. Brooks D. M. (2002). Cross talk between signaling pathways in pathogen defense. *Curr. Opin. Plant Biol.* 5 325–331 10.1016/S1369-5266(02)00275-3 12179966

[B79] Lai Z. Wang F. Zheng Z. Fan B. Chen Z. (2011). A critical role of autophagy in plant resistance to necrotrophic fungal pathogens. *Plant J.* 66 953–968 10.1111/j.1365-313X.2011.04553.x 21395886

[B80] Laurie-Berry N. Joardar V. Street I. H. Kunkel B. N. (2006). The *Arabidopsis thaliana* JASMONATE INSENSITIVE 1 gene is required for suppression of salicylic acid-dependent defenses during infection by *Pseudomonas syringae*. *Mol. Plant Microbe Interact.* 19 789–800 10.1094/MPMI-19-0789 16838791

[B81] Li J. Brader G. Kariola T. Palva E. T. (2006). WRKY70 modulates the selection of signaling pathways in plant defense. *Plant J.* 46 477–491 10.1111/j.1365-313X.2006.02712.x 16623907

[B82] Li J. Brader G. Palva E. T. (2004). The WRKY70 transcription factor: a node of convergence for jasmonate-mediated and salicylate-mediated signals in plant defense. *Plant Cell* 16 319–331 10.1105/tpc.016980 14742872 PMC341906

[B83] Lindeberg M. Cunnac S. Collmer A. (2012). *Pseudomonas syringae* type III effector repertoires: last words in endless arguments. *Trends Microbiol.* 20 199–208 10.1016/j.tim.2012.01.003 22341410

[B84] Liu H. Coulthurst S. J. Pritchard L. Hedley P. E. Ravensdale M. Humphris S. (2008). Quorum sensing coordinates brute force and stealth modes of infection in the plant pathogen Pectobacterium atrosepticum. *PLoS Pathog* 4: e1000093 10.1371/journal.ppat.1000093 PMC2413422 18566662

[B85] Liu Z. Wu Y. Yang F. Zhang Y. Chen S. Xie Q. (2013). BIK1 interacts with PEPRs to mediate ethylene-induced immunity. *Proc. Natl. Acad. Sci. U.S.A.* 110 6205–6210 10.1073/pnas.1215543110 23431184 PMC3625333

[B86] Ma B. Hibbing M. E. Kim H.-S. Reedy R. M. Yedidia I. Breuer J. (2007). Host range and molecular phylogenies of the soft rot enterobacterial genera *Pectobacterium* and *Dickeya*. *Phytopathology* 97 1150–1163 10.1094/PHYTO-97-9-1150 18944180

[B87] Mäe A. Montesano M. Koiv V. Palva E. T. (2001). Transgenic plants producing the bacterial pheromone *N*-acyl-homoserine lactone exhibit enhanced resistance to the bacterial phytopathogen *Erwinia carotovora*. *Mol. Plant Microbe Interact.* 14 1035–1042 10.1094/MPMI.2001.14.9.1035 11551068

[B88] Mattinen L. Tshuikina M. Mäe A. Pirhonen M. (2004). Identification and characterization of Nip, necrosis-inducing virulence protein of *Erwinia carotovora* subsp. *carotovora.* *Mol. Plant Microbe Interact.* 17 1366–1375 10.1094/MPMI.2004.17.12.1366 15597742

[B89] Mauch-Mani B. Mauch F. (2005). The role of abscisic acid in plant–pathogen interactions. *Curr. Opin. Plant Biol.* 8 409–414 10.1016/j.pbi.2005.05.015 15939661

[B90] Melotto M. Underwood W. Koczan J. Nomura K. He S. Y. (2006). Plant stomata function in innate immunity against bacterial invasion. *Cell* 126 969–980 10.1016/j.cell.2006.06.054 16959575

[B91] Mengiste T. (2012). Plant immunity to necrotrophs. *Annu. Rev. Phytopathol.* 50 267–294 10.1146/annurev-phyto-081211-172955 22726121

[B92] Mengiste T. Chen X. Salmeron J. Dietrich R. (2003). The BOTRYTIS SUSCEPTIBLE1 gene encodes an R2R3MYB transcription factor protein that is required for biotic and abiotic stress responses in *Arabidopsis*. *Plant Cell* 15 2551–2565 10.1105/tpc.014167 14555693 PMC280560

[B93] Messiaen J. Read N. D. V. Cutsem P. Trewavas A. J. (1993). Cell wall oligogalacturonides increase cytosolic free calcium in carrot protoplasts. *J. Cell Sci.* 104 365–371

[B94] Miranda J. H. Williams R. W. Kerven G. (2007). Galacturonic acid-induced changes in strawberry plant development in vitro. *In Vitro Cell. Dev. Biol. Plant* 43 639–643 10.1007/s11627-007-9052-7

[B95] Mohr P. G. Cahill D. M. (2003). Abscisic acid influences the susceptibility of *Arabidopsis thaliana* to *Pseudomonas syringae* pv. *tomato* and *Peronospora parasitica*. *Funct. Plant Biol.* 30 461–469 10.1071/FP02231 32689031

[B96] Moloshok T. Pearce G. Ryan C. A. (1992). Oligouronide signaling of proteinase inhibitor genes in plants: structure–activity relationships of Di- and trigalacturonic acids and their derivatives. *Arch. Biochem. Biophys.* 294 731–734 10.1016/0003-9861(92)90748-L 1567229

[B97] Montesano M. Kõiv V. Mäe A. Palva E. T. (2001). Novel receptor-like protein kinases induced by *Erwinia carotovora* and short oligogalacturonides in potato. *Mol. Plant Pathol.* 2 339–346 10.1046/j.1464-6722.2001.00083.x 20573023

[B98] Morris E. R. Powell D. A. Gidley M. J. Rees D. A. (1982). Conformations and interactions of pectins. I. Polymorphism between gel and solid states of calcium polygalacturonate. *J. Mol. Biol.* 155 507–516 10.1016/0022-2836(82)90484-3 7086901

[B99] Moscatiello R. Baldan B. Squartini A. Mariani P. Navazio L. (2012). Oligogalacturonides: novel signaling molecules in Rhizobium-legume communications. *Mol. Plant Microbe Interact.* 25 1387–1395 10.1094/MPMI-03-12-0066-R 22835276

[B100] Moscatiello R. Mariani P. Sanders D. Maathuis F. J. M. (2006). Transcriptional analysis of calcium-dependent and calcium-independent signalling pathways induced by oligogalacturonides. *J. Exp. Bot.* 57 2847–2865 10.1093/jxb/erl043 16868046

[B101] Nabhan S. De Boer S. H. Maiss E. Wydra K. (2012a). *Pectobacterium aroidearum* sp. nov., a soft rot pathogen with preference for monocotyledonous plants. *Int. J. Syst. Evol. Microbiol.* 10.1099/ijs.0.046011-0[Epubaheadofprint]. 23223819

[B102] Nabhan S. Wydra K. Linde M. Debener T. (2012b). The use of two complementary DNA assays, AFLP and MLSA, for epidemic and phylogenetic studies of pectolytic enterobacterial strains with focus on the heterogeneous species *Pectobacterium carotovorum*. *Plant Pathol*. 61 498–508 10.1111/j.1365-3059.2011.02546.x

[B103] Nadarasah G. Stavrinides J. (2011). Insects as alternative hosts for phytopathogenic bacteria. *FEMS Microbiol. Rev.* 35 555–575 10.1111/j.1574-6976.2011.00264.x 21251027

[B104] Niks R. E. Marcel T. C. (2009). Nonhost and basal resistance: how to explain specificity? *New Phytol.* 182 817–828 10.1111/j.1469-8137.2009.02849.x 19646067

[B105] Norman C. Vidal S. Palva E. T. (1999). Oligogalacturonide-mediated induction of a gene involved in jasmonic acid synthesis in response to the cell-wall-degrading enzymes of the plant pathogen *Erwinia carotovora*. *Mol. Plant Microbe Interact.* 12 640–644 10.1094/MPMI.1999.12.7.640 10478482

[B106] Norman-Setterblad C. Vidal S. Palva E. T. (2000). Interacting signal pathways control defense gene expression in *Arabidopsis* in response to cell wall-degrading enzymes from *Erwinia carotovora*. *Mol. Plant Microbe Interact.* 13 430–438 10.1094/MPMI.2000.13.4.430 10755306

[B107] Nykyri J. Niemi O. Koskinen P. Nokso-Koivisto J. Pasanen M. Broberg M. (2012). Revised phylogeny and novel horizontally acquired virulence determinants of the model soft rot phytopathogen *Pectobacterium wasabiae* SCC3193. *PLoS Pathog.* 8: e1003013 10.1371/journal.ppat.1003013 PMC3486870 23133391

[B108] O’Donnell P. J. Calvert C. Atzorn R. Wasternack C. Leyser H. M. O. Bowles D. J. (1996). Ethylene as a signal mediating the wound response of tomato plants. *Science* 274 1914–1917 10.1126/science.274.5294.1914 8943205

[B109] Palva T. K. Holmström K. O. Heino P. Palva E. T. (1993). Induction of plant defense response by exoenzymes of *Erwinia carotovora* subsp. *carotovora. Mol. Plant Microbe Interact.* 6 190–196 10.1094/MPMI-6-190

[B110] Palva T. K. Hurtig M. Saindrenan P. Palva E. T. (1994). Salicylic acid induced resistance to *Erwinia carotovora* subsp. *carotovora* in tobacco. *Mol. Plant Microbe Interact.* 7 356–363 10.1094/MPMI-7-0356

[B111] Park S.-W. Kaimoyo E. Kumar D. Mosher S. Klessig D. F. (2007). Methyl salicylate is a critical mobile signal for plant systemic acquired resistance. *Science* 318 113–116 10.1126/science.1147113 17916738

[B112] Park T.-H. Choi B.-S. Choi A.-Y. Choi I.-Y. Heu S. Park B.-S. (2012). Genome sequence of *Pectobacterium carotovorum* subsp. *carotovorum* strain PCC21, a pathogen causing soft rot in Chinese cabbage. *J. Bacteriol.* 194 6345–6346 10.1128/JB.01583-12 23105077 PMC3486416

[B113] Parker J. E. (2003). Plant recognition of microbial patterns. *Trends Plant Sci.* 8 245–247 10.1016/S1360-1385(03)00105-5 12818655

[B114] Pena-Cortés H. Albrecht T. Prat S. Weiler E. W. Willmitzer L. (1993). Aspirin prevents wound-induced gene expression in tomato leaves by blocking jasmonic acid biosynthesis. *Planta* 191 123–128 10.1007/BF00240903

[B115] Pérombelon M. C. M. (2002). Potato diseases caused by soft rot erwinias: an overview of pathogenesis. *Plant Pathol.* 51 1–12 10.1046/j.0032-0862.2001.Shorttitle.doc.x

[B116] Perombelon M. C. M. Kelman A. (1980). Ecology of the soft rot erwinias. *Annu. Rev. Phytopathol.* 18 361–387 10.1146/annurev.py.18.090180.002045

[B117] Pieterse C. M. J. Leon-Reyes A. Van der Ent S. Van Wees S. C. M. (2009). Networking by small-molecule hormones in plant immunity. *Nat. Chem. Biol.* 5 308–316 10.1038/nchembio.164 19377457

[B118] Pirhonen M. Flego D. Heikinheimo R. Palva E. T. (1993). A small diffusible signal molecule is responsible for the global control of virulence and exoenzyme production in the plant pathogen *Erwinia carotovora*. *EMBO J.* 12 2467–2476 8508772 10.1002/j.1460-2075.1993.tb05901.x PMC413482

[B119] Pitman A. R. Harrow S. A. Visnovsky S. B. (2009). Genetic characterisation of Pectobacterium wasabiae causing soft rot disease of potato in New Zealand. *Eur. J. Plant Pathol.* 126 423–435 10.1007/s10658-009-9551-y

[B120] Pitman A. R. Wright P. J. Galbraith M. D. Harrow S. A. (2008). Biochemical and genetic diversity of pectolytic enterobacteria causing soft rot disease of potatoes in New Zealand. *Australas. Plant Pathol* 37 559 10.1071/AP08056

[B121] Po-Wen C. Singh P. Zimmerli L. (2013). Priming of the *Arabidopsis* pattern-triggered immunity response upon infection by necrotrophic *Pectobacterium carotovorum* bacteria. *Mol. Plant Pathol.* 14 58–70 10.1111/j.1364-3703.2012.00827.x 22947164 PMC6638802

[B122] Qi L. Yan J. Li Y. Jiang H. Sun J. Chen Q. (2012). *Arabidopsis* thaliana plants differentially modulate auxin biosynthesis and transport during defense responses to the necrotrophic pathogen Alternaria brassicicola. *New Phytol.* 195 872–882 10.1111/j.1469-8137.2012.04208.x 22731664

[B123] Randoux B. Renard-Merlier D. Duyme F. Sanssené J. Courtois J. Durand R. (2009). Oligogalacturonides induce resistance in wheat against powdery mildew. *Commun. Agric. Appl. Biol. Sci.* 74 681–685 20222550

[B124] Randoux B. Renard-Merlier D. Mulard G. Rossard S. Duyme F. Sanssené J. (2010). Distinct defenses induced in wheat against powdery mildew by acetylated and nonacetylated oligogalacturonides. *Phytopathology* 100 1352–1363 10.1094/PHYTO-03-10-0086 20684658

[B125] Rantakari A. Virtaharju O. Vähä miko S. Taira S. Palva E. T. Saarilahti H. T. (2001). Type III secretion contributes to the pathogenesis of the soft-rot pathogen *Erwinia carotovora*: partial characterization of the hrp gene cluster. *Mol. Plant Microbe Interact.* 14 962–968 10.1094/MPMI.2001.14.8.962 11497468

[B126] Rasul S. Dubreuil-Maurizi C. Lamotte O. Koen E. Poinssot B. Alcaraz G. (2012). Nitric oxide production mediates oligogalacturonide-triggered immunity and resistance to *Botrytis cinerea* in *Arabidopsis thaliana*. *Plant Cell Environ.* 35 1483–1499 10.1111/j.1365-3040.2012.02505.x 22394204

[B127] Records A. R. (2011). The type VI secretion system: a multipurpose delivery system with a phage-like machinery. *Mol. Plant Microbe Interact.* 24 751–757 10.1094/MPMI-11-10-0262 21361789

[B128] Ridley B. L. O’Neill M. A. Mohnen D. (2001). Pectins: structure, biosynthesis, and oligogala{-}cturonide-related signaling. *Phytochemistry* 57 929–967 10.1016/S0031-9422(01)00113-3 11423142

[B129] Robatzek S. Chinchilla D. Boller T. (2006). Ligand-induced endocytosis of the pattern recognition receptor FLS2 in *Arabidopsis*. *Genes Dev.* 20 537–542 10.1101/gad.366506 16510871 PMC1410809

[B130] Robert-Seilaniantz A. Grant M. Jones J. D. G. (2011). Hormone crosstalk in plant disease and defense: more than just jasmonate-salicylate antagonism. *Annu. Rev. Phytopathol.* 49 317–343 10.1146/annurev-phyto-073009-114447 21663438

[B131] Ross J. R. Nam K. H. D’Auria J. C. Pichersky E. (1999). *S*-Adenosyl-l-methionine:salicylic acid carboxyl methyltransferase, an enzyme involved in floral scent production and plant defense, represents a new class of plant methyltransferases. *Arch. Biochem. Biophys.* 367 9–16 10.1006/abbi.1999.1255 10375393

[B132] Russell A. B. Hood R. D. Bui N. K. LeRoux M. Vollmer W. Mougous J. D. (2011). Type VI secretion delivers bacteriolytic effectors to target cells. *Nature* 475 343–347 10.1038/nature10244 21776080 PMC3146020

[B133] Saarilahti H. T. Heino P. Pakkanen R. Kalkkinen N. Palva I. Palva E. T. (1990). Structural analysis of the pehA gene and characterization of its protein product, endopolygalacturonase, of *Erwinia carotovora* subspecies *carotovora*. *Mol. Microbiol.* 4 1037–1044 10.1111/j.1365-2958.1990.tb00676.x 2215212

[B134] Salmond G. P. Bycroft B. W. Stewart G. S. Williams P. (1995). The bacterial “enigma”: cracking the code of cell-cell communication. *Mol. Microbiol.* 16 615–624 10.1111/j.1365-2958.1995.tb02424.x 7476157

[B135] Samson R. Legendre J. B. Christen R. Fischer-Le Saux M. Achouak W. Gardan L. (2005). Transfer of *Pectobacterium chrysanthemi* (Burkholder et al. 1953) Brenner et al. 1973 and *Brenneria paradisiaca* to the genus *Dickeya* gen. nov. as *Dickeya chrysanthemi* comb. nov. and *Dickeya paradisiaca* comb. nov. and delineation of four novel species, *Dickeya dadantii* sp. nov., *Dickeya dianthicola* sp. nov., *Dickeya dieffenbachiae* sp. nov. and *Dickeya zeae* sp. nov *Int. J. Syst. Evol. Microbiol.* 55 1415–1427 10.1099/ijs.0.02791-0 16014461

[B136] Savatin D. V. Ferrari S. Sicilia F. De Lorenzo G. (2011). Oligogalacturonide-auxin antagonism does not require posttranscriptional gene silencing or stabilization of auxin response repressors in *Arabidopsis*. *Plant Physiol.* 157 1163–1174 10.1104/pp.111.184663 21880931 PMC3252154

[B137] Schacht T. Unger C. Pich A. Wydra K. (2011). Endo- and exopolygalacturonases of *Ralstonia solanacearum* are inhibited by polygalacturonase-inhibiting protein (PGIP) activity in tomato stem extracts. *Plant Physiol. Biochem.* 49 377–387 10.1016/j.plaphy.2011.02.001 21367611

[B138] Schwarz S. West T. E. Boyer F. Chiang W.-C. Carl M. A. Hood R. D. (2010). Burkholderia type VI secretion systems have distinct roles in eukaryotic and bacterial cell interactions. *PLoS Pathog.* 6: e1001068 10.1371/journal.ppat.1001068 PMC2928800 20865170

[B139] Segonzac C. Zipfel C. (2011). Activation of plant pattern-recognition receptors by bacteria. *Curr. Opin. Microbiol.* 14 54–61 10.1016/j.mib.2010.12.005 21215683

[B140] Simpson S. D. Ashford D. A. Harvey D. J. Bowles D. J. (1998). Short chain oligogalacturonides induce ethylene production and expression of the gene encoding aminocyclopropane 1-carboxylic acid oxidase in tomato plants. *Glycobiology* 8 579–583 10.1093/glycob/8.6.579 9592124

[B141] Spoel S. H. Koornneef A. Claessens S. M. C. Korzelius J. P. Van Pelt J. A. Mueller M. J. (2003). NPR1 modulates cross-talk between salicylate- and jasmonate-dependent defense pathways through a novel function in the cytosol. *Plant Cell* 15 760–770 10.1105/tpc.009159 12615947 PMC150028

[B142] Spoel S. H. Loake G. J. (2011). Redox-based protein modifications: the missing link in plant immune signalling. *Curr. Opin. Plant Biol.* 14 358–364 10.1016/j.pbi.2011.03.007 21454121

[B143] Tao Y. Xie Z. Chen W. Glazebrook J. Chang H.-S. Han B. (2003). Quantitative nature of *Arabidopsis* responses during compatible and incompatible interactions with the bacterial pathogen *Pseudomonas syringae*. *Plant Cell* 15 317–330 10.1105/tpc.007591 12566575 PMC141204

[B144] Thain J. F. Doherty H. M. Bowles D. J. Wildon D. C. (1990). Oligosaccharides that induce proteinase inhibitor activity in tomato plants cause depolarization of tomato leaf cells. *Plant Cell Environ.* 13 569–574 10.1111/j.1365-3040.1990.tb01074.x

[B145] Ton J. Flors V. Mauch-Mani B. (2009). The multifaceted role of ABA in disease resistance. *Trends Plant Sci.* 14 310–317 10.1016/j.tplants.2009.03.006 19443266

[B146] Toth I. K. Bell K. S. Holeva M. C. Birch P. R. J. (2003). Soft rot erwiniae: from genes to genomes. *Mol. Plant Pathol.* 4 17–30 10.1046/j.1364-3703.2003.00149.x 20569359

[B147] Toth I. K. Birch P. R. J. (2005). Rotting softly and stealthily. *Curr. Opin. Plant Biol.* 8 424–429 10.1016/j.pbi.2005.04.001 15970273

[B148] Toth I. K. Pritchard L. Birch P. R. J. (2006). Comparative genomics reveals what makes an enterobacterial plant pathogen. *Annu. Rev. Phytopathol.* 44 305–336 10.1146/annurev.phyto.44.070505.143444 16704357

[B149] Toth I. K. van der Wolf J. M. Saddler G. Lojkowska E. Hélias V. Pirhonen M. (2011). Dickeya species: an emerging problem for potato production in Europe. *Plant Pathol.* 60 385–399 10.1111/j.1365-3059.2011.02427.x

[B150] Uppalapati S. R. Ishiga Y. Wangdi T. Kunkel B. N. Anand A. Mysore K. S. (2007). The phytotoxin coronatine contributes to pathogen fitness and is required for suppression of salicylic acid accumulation in tomato inoculated with *Pseudomonas syringae* pv. *tomato* DC3000. *Mol. Plant Microbe Interact.* 20 955–965 10.1094/MPMI-20-8-0955 17722699

[B151] van Kan J. A. L. (2006). Licensed to kill: the lifestyle of a necrotrophic plant pathogen. *Trends Plant Sci.* 11 247–253 10.1016/j.tplants.2006.03.005 16616579

[B152] Verslues P. E. Zhu J.-K. (2005). Before and beyond ABA: upstream sensing and internal signals that determine ABA accumulation and response under abiotic stress. *Biochem. Soc. Trans.* 33 375–379 10.1042/BST0330375 15787610

[B153] Vidal S. Ponce de Leon I. Denecke J. Palva E. T. (1997). Salicylic acid and the plant pathogen *Erwinia carotovora* induce plant defense genes by antagonistic pathways. *Plant J.* 115–123 10.1046/j.1365-313X.1997.11010115.x

[B154] Weber J. Olsen O. Wegener C. von Wettstein D. (1996). Digalacturonates from pectin degradation induce tissue responses against potato soft rot. *Physiol. Mol. Plant Pathol.* 48 389–401 10.1006/pmpp.1996.0031

[B155] Wegener C. Bartling S. Olsen O. Weber J. von Wettstein D. (1996). Pectate lyase in transgenic potatoes confers pre-activation of defence against *Erwinia carotovora*. *Physiol. Mol. Plant Pathol.* 49 359–376 10.1006/pmpp.1996.9998

[B156] Winslow C. E. Broadhurst J. Buchanan R. E. Krumwiede C. Rogers L. A. Smith G. H. (1920). The families and genera of the bacteria: final report of the Committee of the Society of American Bacteriologists on Characterization and Classification of Bacterial Types. *J. Bacteriol.* 5 191–229 16558872 10.1128/jb.5.3.191-229.1920 PMC378870

[B157] Yang S. Zhang Q. Guo J. Charkowski A. O. Glick B. R. Ibekwe A. M. (2007). Global effect of indole-3-acetic acid biosynthesis on multiple virulence factors of *Erwinia chrysanthemi* 3937. *Appl. Environ. Microbiol.* 73 1079–1088 10.1128/AEM.01770-06 17189441 PMC1828641

[B158] Zeng W. He S. Y. (2010). A prominent role of the flagellin receptor FLAGELLIN-SENSING2 in mediating stomatal response to Pseudomonas syringae pv tomato DC3000 in *Arabidopsis*. *Plant Physiol.* 153 1188–1198 10.1104/pp.110.157016 20457804 PMC2899927

[B159] Zheng J. Ho B. Mekalanos J. J. (2011). Genetic analysis of anti-amoebae and anti-bacterial activities of the type VI secretion system in *Vibrio cholerae*. *PLoS ONE* 6: e23876. 10.1371/journal.pone.0023876 PMC3166118 21909372

[B160] Zheng X.-Y. Spivey N. W. Zeng W. Liu P.-P. Fu Z. Q. Klessig D. F. (2012). Coronatine promotes *Pseudomonas syringae* virulence in plants by activating a signaling cascade that inhibits salicylic acid accumulation. *Cell Host Microbe* 11 587–596 10.1016/j.chom.2012.04.014 22704619 PMC3404825

[B161] Zipfel C. Robatzek S. (2010). Pathogen-associated molecular pattern-triggered immunity: Veni, Vidi…? *Plant Physiol.* 154 551–554 10.1104/pp.110.161547 20921183 PMC2949051

[B162] Zipfel C. Robatzek S. Navarro L. Oakeley E. J. Jones J. D. G. Felix G. (2004). Bacterial disease resistance in *Arabidopsis* through flagellin perception. *Nature* 428 764–767 10.1038/nature02485 15085136

